# The Personalized Inherited Signature Predisposing to Non-Small-Cell Lung Cancer in Non-Smokers

**DOI:** 10.3390/cancers16162887

**Published:** 2024-08-20

**Authors:** Viola Bianca Serio, Diletta Rosati, Debora Maffeo, Angela Rina, Marco Ghisalberti, Cristiana Bellan, Ottavia Spiga, Francesca Mari, Maria Palmieri, Elisa Frullanti

**Affiliations:** 1 Cancer Genomics & Systems Biology Laboratory, University of Siena, 53100 Siena, Italy; viola.serio@dbm.unisi.it (V.B.S.); diletta.rosati2@unisi.it (D.R.); debora.maffeo@dbm.unisi.it (D.M.); maria.palmieri@dbm.unisi.it (M.P.); 2 Med Biotech Hub and Competence Centre, Department of Medical Biotechnologies, University of Siena, 53100 Siena, Italy; angela.rina@student.unisi.it (A.R.); francesca.mari@unisi.it (F.M.); 3 Thoracic Surgery Unit, Azienda Ospedaliera Universitaria Senese, 53100 Siena, Italy; ghisamarco@gmail.com; 4 Department of Medical Biotechnology, Section of Pathology, University of Siena, 53100 Siena, Italy; cristiana.bellan@unisi.it; 5 Department of Biotechnology, Chemistry and Pharmacy, University of Siena, 53100 Siena, Italy; ottavia.spiga@unisi.it; 6 UOC Laboratorio di Assistenza e Ricerca Traslazionale, Azienda Ospedaliero-Universitaria Senese, 53100 Siena, Italy

**Keywords:** whole-exome sequencing, lung cancer susceptibility, germline variants, next-generation sequencing, oligogenic model

## Abstract

**Simple Summary:**

Building on the idea of a germline oligogenic origin of lung cancer, we performed WES of DNA from patients’ peripheral blood and their unaffected sibs. Filtering for rare variants and potentially damaging effects, we identified 40 deleterious variants mapping in genes previously associated with cancer exclusively identified in patients. Transcriptome profiling on both tumor and normal lung tissues revealed that, among the selected mutated genes, 16 variants mapping in 16 genes were either down- or upregulated in cancer specimens. Among the downregulated genes, 9 variants in 9 genes carried the mutated allele suggesting a loss of heterozygosity. Notably, the group of mutated genes was unique for each patient, pinpointing to a “private” oligogenic germline signature. In the era of precision medicine, this report emphasizes the importance of an “omic” approach to uncover an oligogenic germline signature underlying cancer development and identify suitable therapeutic targets.

**Abstract:**

Lung cancer (LC) continues to be an important public health problem, being the most common form of cancer and a major cause of cancer deaths worldwide. Despite the great bulk of research to identify genetic susceptibility genes by genome-wide association studies, only few loci associated to nicotine dependence have been consistently replicated. Our previously published study in few phenotypically discordant sib-pairs identified a combination of germline truncating mutations in known cancer susceptibility genes in never-smoker early-onset LC patients, which does not present in their healthy sib. These results firstly demonstrated the presence of an oligogenic combination of disrupted cancer-predisposing genes in non-smokers patients, giving experimental support to a model of a “private genetic epidemiology”. Here, we used a combination of whole-exome and RNA sequencing coupled with a discordant sib’s model in a novel cohort of pairs of never-smokers early-onset LC patients and in their healthy sibs used as controls. We selected rare germline variants predicted as deleterious by CADD and SVM bioinformatics tools and absent in the healthy sib. Overall, we identified an average of 200 variants per patient, about 10 of which in cancer-predisposing genes. In most of them, RNA sequencing data reinforced the pathogenic role of the identified variants showing: (i) downregulation in LC tissue (indicating a “second hit” in tumor suppressor genes); (ii) upregulation in cancer tissue (likely oncogene); and (iii) downregulation in both normal and cancer tissue (indicating transcript instability). The combination of the two techniques demonstrates that each patient has an average of six (with a range from four to eight) private mutations with a functional effect in tumor-predisposing genes. The presence of a unique combination of disrupting events in the affected subjects may explain the absence of the familial clustering of non-small-cell lung cancer. In conclusion, these findings indicate that each patient has his/her own “predisposing signature” to cancer development and suggest the use of personalized therapeutic strategies in lung cancer.

## 1. Introduction

Lung cancer (LC) is an important public health problem and a major cause of cancer death worldwide [1]. Despite the strong relationship to tobacco smoking, less than 1 out of 5 heavy smokers develop lung cancer [2], whereas 25% of patients with lung cancer never smoked [3], suggesting a role for genetic factors in the individual susceptibility to lung cancer. Thus far, no specific environmental or genetic risk factors have been detected in these individuals.

Previous genome-wide association studies (GWASs) have identified up to 45 susceptibility loci for lung cancer in population-based series, mainly including smokers [4,5,6,7,8,9,10,11,12,13,14,15]. On the other hand, several studies found the rare germline T790M mutation in *EGFR* correlated with the familial clustering of lung cancer [16,17,18,19,20,21,22,23]. The *EGFR* is a major cancer-predisposition gene with an estimated 31% risk of lung cancer development in non-smoking carriers [19]. However, the *EGFR* T790M mutation is weakly oncogenic and became significantly enhanced when associated with a second activating mutation [18], more frequently with the female sex and non-smoking status. Nevertheless, today, there is a need to better understand the genetic factors associated with lung cancer in never-smokers’ patients in non-predisposed families.

In this frame, using the whole-exome sequencing approach, in a previous study analyzing discordant sib-pairs, we identified a combination of germline disruptive mutations in known cancer susceptibility genes in never-smokers with lung adenocarcinoma (ADCA) of early onset, that were not present in their healthy control sibs [24]. These results demonstrated that each affected subject had an oligogenic combination of disrupted cancer-predisposing genes. This evidence gives experimental support to a model of “private genetic epidemiology” for lung cancer susceptibility that has previously only been hypothesized. The oligogenic nature of the model may therefore explain the non-heritability of the condition.

In the present study, we used a combination of genetic technical tools (whole-exome sequencing analysis and RNA sequencing) coupled with a pedigree model in discordant pairs of non-smokers with lung cancer of early onset. Healthy sibs were used as controls. We identified in affected subjects a unique combination of private “predisposing signatures” that further confirms and exploits the oligogenic model of the disease.

## 2. Materials and Methods

### 2.1. Study Design and Samples

This study analyzed four non-smoker lung cancer patients with lung cancer (cases), who underwent lung lobectomy in the Thoracic Surgery Unit at the Azienda Ospedaliero-Universitaria Senese (AOUS, Siena, Italy), in comparison with their healthy sibs (controls). The genetic comparison of discordant sibs, that share 50% of the genome, facilitates the identification of variants associated with lung cancer susceptibility. For each patient, formalin-fixed paraffin-embedded (FFPE) samples of tumoral and non-tumoral lung tissues were obtained from the Pathology Unit of the recruiting hospital and analyzed through whole-exome sequencing and RNA sequencing (RNA-seq). 

Each patient and sib signed the informed consent declaration for the use of their biological samples and clinical data for research purposes. The study protocol was approved by the Institutional Ethical Committees. Information on histological diagnoses (by the Pathology Unit) was retrieved from the clinical records. 

### 2.2. DNA and RNA Extraction

Genomic DNA of cases and controls was isolated from EDTA peripheral blood using QIAamp DNA Blood Kit (Qiagen^®^, Hilden, Germany) according to the manufacturer’s protocol. DNA was extracted from FFPE lung tumoral and non-tumoral tissue samples using MagCore^®^ Genomic DNA FFPE One-Step Kit for MagCore^®^ System (Diatech Pharmacogenetics srl, Jesi, Ancona, Italy). RNA was extracted from FFPE lung tumoral and non-tumoral tissue samples using High Pure FFPE-Tissue RNA Isolation Kit (Roche, Basel, Switzerland) following the manufacturer’s instructions. RNA samples (1 µg) were processed to remove ribosomal RNA (rRNA) using Ribo-Zero rRNA Removal Kit for Human samples (Illumina, Grand Island, NE, USA) following the manufacturer’s instructions. RNA integrity was verified using the Agilent Eukaryote Total RNA Nano Kit (Agilent Technologies, Palo Alto, CA, USA) on Agilent2100 Bioanalyzer (Agilent Technologies). Both DNA and RNA were quantified by spectrophotometry (ND-2000c; NanoDrop Products, Wilmington, DE, USA) and Qubit^®^ Fluorometer with Qubit^®^ dsDNA HS Assay and Qubit^®^ RNA HS Assay Kits (Life Technologies, Carlsbad, CA, USA), respectively.

### 2.3. Whole-Exome Sequencing and Data Analysis

Whole-exome sequencing was performed using the Illumina Nextseq 550 on genomic DNA samples (500 ng) of cases and controls and tumor tissues as previously described [25]. After variant annotation against external datasets, including 1000 genomes [(http://www.1000genomes.org/), accessed on 19 February 2024] and dbSNP, in order to identify susceptibility variants, we selected for rare variants (minor allele frequency—MAF ≤ 0.01) with a potentially damaging effect and with a functional link to cancer development. Additional filtering procedures were thus implemented for: (i) retrieving exonic rare variants with a potential detrimental impact on protein function, i.e., truncating frameshift insertion and deletion and nonsense variants predicted as deleterious that were present in affected but not in unaffected sibs and vice versa; and (ii) identifying somatic mutations present in tumor tissues. 

### 2.4. RNA Sequencing and Data Analysis

RNA sequencing was performed using Illumina HiSeq2500 platform (Illumina), in a 2 × 100 bp paired-end (PE) configuration in High Output mode (V4 chemistry), with a total of at least 250 million reads per lane. After quality check, RNA (50 ng) was used to prepare sample libraries. Sequencing library construction included these steps: library construction using Illumina TruSeq RNA Sample Pre Kit (Illumina), library purification using Beckman AMPure XP beads (Beckman Coulter s.r.l., Milan, Italy), insert fragments test using Agilent High Sensitivity DNA Kit on Agilent 2100 Bioanalyzer (Agilent Technologies), quantitative analysis of library (ABI 7500 real time PCR instrument; KAPA SYBR green fast universal 2 9 qPCR master mix. GRN), and cBOT automatic cluster (TruSeq PE Cluster Kit v3-cBotHS). Post-library quality controls were performed using the Agilent RNA 6000 Nano kit (Agilent Technologies) on Agilent2100 Bioanalyzer (Agilent Technologies) and Qubit^®^ Fluorometer (Life Technologies). Libraries were then loaded on HiSeq2500 sequencing platform (Illumina) and sequenced using 2 × 100 bp pair-end High Output Mode (V4 chemistry) per lane. The reads generated on the HiSeq2500 were provided under FASTQ format. 

Sequence reads in FASTQ format were processing using the Fastqc software [(http://www.bioinformatics.babraham.ac.uk/projects/fastqc/), accessed on 4 March 2024, Version 0.12.0] for data quality check and removing excess adaptors to obtain high-quality and clean reads. The high-quality reads were aligned to the GRCh38/hg38 reference human genome [(ftp://jgi-psf.org/pub/compgen/phytozome/v9.0/Ptrichocarpa/assembly/Ptrichocarpa_210.fa.gz), accessed on 4 March 2024] using the TopHat software (version 2.1.1) [26]. Transcript assembling and expression quantification were carried out using Cufflinks (version 2) [27]. Gene expression was expressed as fragments per kilo-base transcript per million mapped reads (FPKM) value [28]. This normalized value was used for visualization on a genome browser [(http://genome.ucsc.edu/), accessed on 20 March 2024] [29], as well as to compare read coverage between and throughout different genes. Statistical analysis was performed to compute the mean FPKM level with the associated P-value for lung normal tissues together with the mean FPKM level with the associated P-value for lung cancer tissues. Cuffdiff tool from Cufflinks was used to identify differentially expressed genes [30]. Potential gene fusion events were detected by TopHat-fusion [31] with spanning reads ≥10 in cancer and normal tissue. The cancer-specific fusion genes were obtained by excluding the fusion genes that were also identified in distant normal tissue. Gene Ontology (GO) and pathways analyses were performed using the Database for Annotation, Visualization, and Integrated Discovery functional annotation tool [(DAVID Bioinformatics Resources v2024q1, https://david.ncifcrf.gov), accessed on 16 April 2024].

## 3. Results

### 3.1. Exome Analysis of Constitutive DNA

We carried out the whole-exome sequencing of DNA from blood tissue in four discordant sib pairs (Table 1). Patients had early-onset lung cancer in the absence of smoking history, and we used as controls their unaffected sibling. Exome sequencing generated a mean of 28,960,442 reads per sample with a mean read length of 160 bp.

**Figure 1:**
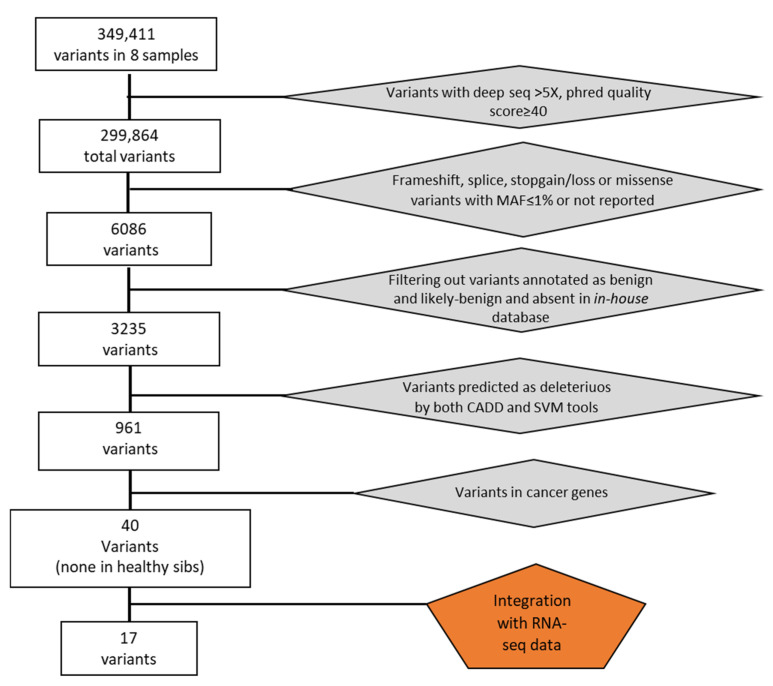
Flowchart illustrating filtering process and variants selection.

Overall, we identified a total of 349,411 genetic variants in eight samples. After removing variants with low coverage and filtering for exonic mutations with MAF ≤ 0.01 or not reported, we obtained 6086 total variants (Figure 1). We then filtered by excluding variants with clinical significance as “benign” or “likely benign” and present in an in-house database of variants identifying 3235 variants. Among them, we selected deleterious variants applied to the Combined Annotation Dependent Depletion (CADD) and the MetaSVM (Support Vector Machine) bioinformatics tools. In this way, we obtained 961 potential deleterious variants of which 370 were non-synonymous and 591 were truncating variants (insertions and deletions (indels) causing exonic frameshifts, and nonsense mutations leading to truncated proteins). 

Out of these, 40 variants mapped in genes that have been found previously associated with cancer of lung or other tissues and, interestingly, all of these were present exclusively in the affected sib (Table 2). The validation of these variants was carried out using a custom next-generation sequencing (NGS) panel for the Ion PGM sequencer (Life Technologies) and led to the confirmation of 40 variants probably responsible for lung cancer susceptibility in our cases. All the 40 sequence variants identified were in a heterozygous state. No common variants to all four cases were found. However, three variants were common to two out of four patients (*ANGPLT4*, *CARS*, and *ESRRA*). The two variants in Angiopoietin-Like 4 (*ANGPLT4*) and Estrogen-Related Receptor Alpha (*ESRRA*) genes were common to case 1 and case 4, while the same variant in the Cysteinyl-TRNA Synthetase (*CARS*) gene was found in case 3 and case 4. Each of the 40 variants were also present in the relative lung tumor tissue in the heterozygous state. 

### 3.2. Somatic Mutations in Tumor Lung Tissues

**Figure 2:**
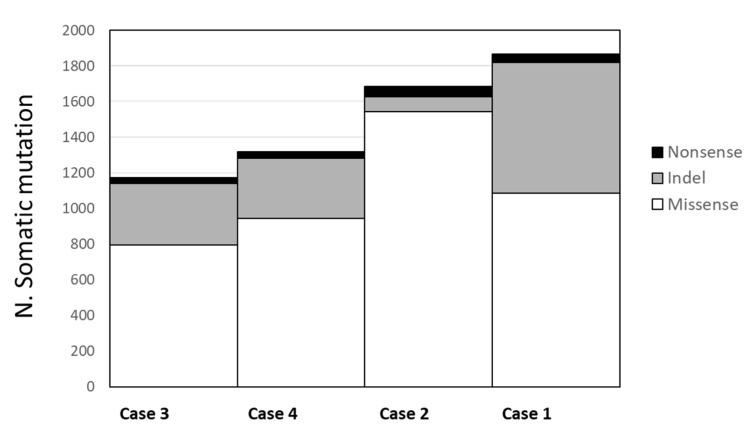
Average number of somatic mutations in tumor lung tissues. Missense mutations have a numerical range from 797 to 1542 (in white); indel mutations range from 85 to 735 (in gray); and, in black, the nonsense mutations have a range from 32 to 56.

In the four tumor lung samples, we identified an average of 2.568 somatic mutations per tumor (ranging from 2.169 to 3.348). When we limited the mutations in the coding regions, the average number of mutations in each tumor was 1.510 (range 1.172–1.865), among which an average of 1092 (range 797–1542) were missense, 374 (range 85–735) were frameshift ins/del, and 43 (range 32–56) were nonsense mutations (Figure 2). The number of mutations was not associated with clinical and pathological variables (stage and age at diagnosis). No recurrent mutations, such as mutations in *EGFR*, *KRAS*, or *AKT* genes, were present in our tumor samples.

### 3.3. RNA-Seq Analysis of Tumors and Non-Involved Lung Tissues

To detect differences in the expression level, splicing pattern, and/or polyadenylation sites that could both help the understanding of the functional role of germline variants in the development of lung cancer and shed light on the pathogenic mechanisms, we performed an RNA sequencing (RNA-seq) analysis of RNA extracted from both the FFPE tumor and non-involved lung tissues of the four lung cancer patients. RNA-seq generated a mean of 73,865,049 reads per sample and 91.07% of bases sequenced above the Q30 quality score (Supplementary Table S1). 

**Figure 3:**
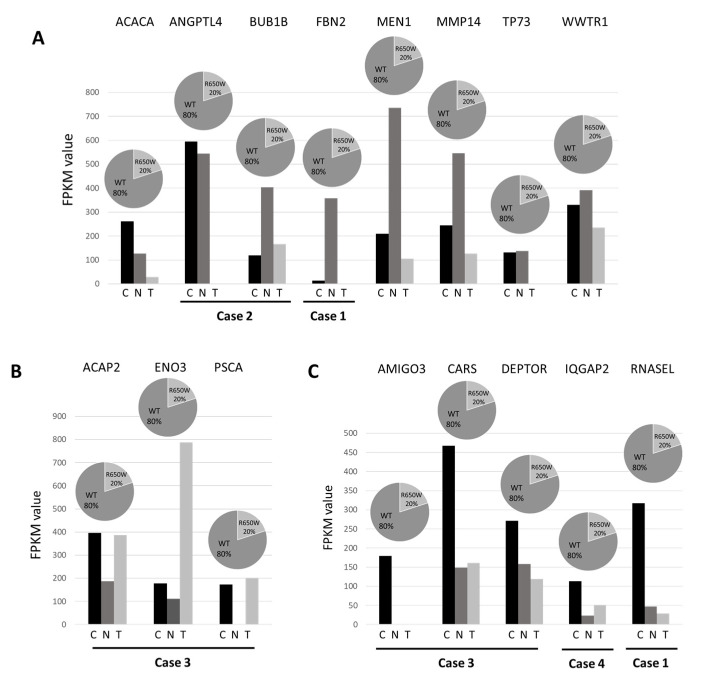
RNA transcript levels in normal and tumor tissue pairs of 16 candidate germline mutated genes. RNA levels were expressed as FPKM value in tumor tissue (T), in the normal counterpart (N) and in the group of normal tissues (C). *ACACA*, *ANGPTL4*, *BUB1B*, *FBN2*, *MEN1*, *MMP14*, *TP73*, and *WWTR1* showed a downregulation in lung cancer tissue (panel (**A**)); *ACAP2*, *ENO3*, and *PSCA* showed an upregulation in lung cancer tissue (panel (**B**)); *AMIGO3*, *CARS*, *DEPTOR*, *IQGAP2*, and *RNASEL* were downregulated in both normal and cancer tissue (panel (**C**)).

To assess the potential functional role of the 40 germline variants identified by whole-exome sequencing (WES) and predicted as deleterious by bioinformatics tools, we combined the results from the WES experiments with the respective expression profile from RNA-seq. In 16 variants mapping in 16 genes, the RNA sequencing data reinforced the pathogenic role of the identified variants showing three different effects (Figure 3). Firstly, eight genes [Acetyl-CoA carboxylase alpha (*ACACA*), Angiopoietin like 4 *(ANGPTL4)*, BUB1 mitotic checkpoint serine/threonine kinase B (*BUB1B*), Fibrillin 2 (*FBN2*), Menin 1 (*MEN1*), Matrix metallopeptidase 14 (*MMP14*), Tumor protein 73 (*TP73),* and WW domain-containing transcription regulator protein 1 (*WWTR1*)] showed a downregulation in lung cancer tissue, indicating a possible “second hit” in tumor suppressor genes responsible for gene inactivation (Figure 3A). Secondly, three genes [ArfGAP With Coiled-Coil, Ankyrin Repeat And PH Domains 2 (*ACAP2)*, Enolase3 (*ENO3)*, and prostate stem cell antigen (*PSCA*)] showed an upregulation in lung cancer tissue, suggesting their role as an oncogene (Figure 3B). Lastly, five remaining genes [Adhesion Molecule With Ig Like Domain 3 (*AMIGO3*), Cysteinyl-tRNA synthetase (*CARS*), DEP Domain Containing MTOR Interacting Protein (*DEPTOR*), IQ Motif Containing GTPase Activating Protein 2 (*IQGAP2*), and Ribonuclease L (*RNASEL*)] were downregulated in both normal and cancer tissue, indicating a possible transcript instability (Figure 3C). In addition, a nonsense variant that was predicted as deleterious in the putative tumor suppressor URI1 Prefoldin Like Chaperone (*URI1*) gene, although it was not associated with changes in mRNA levels, was also retained in the panel of candidate genes.

**Figure 4:**
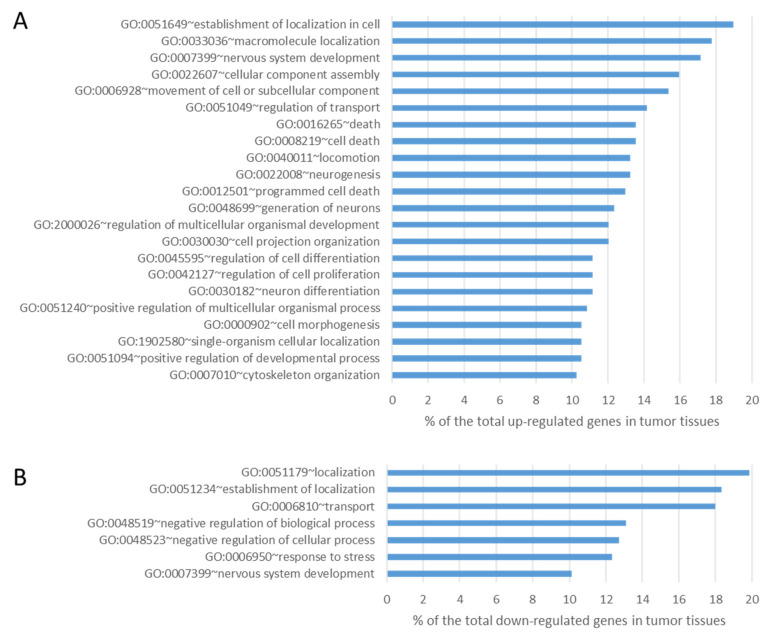
Results of GO analysis of upregulated (panel (**A**)) and downregulated (panel (**B**)) transcripts in lung tumor tissues compared with lung normal tissues (all components with >10% and *p*-value < 0.05).

The data obtained from tumor tissues were then compared with those from normal tissues in order to identify differences in the expression level using well-established and accepted analysis tools such as Cufflink-Cuffdiff [30]. Cuffdiff differential expression analysis identified 315 genes significantly downregulated (fold ≤ −2 change, *p*-value ≤ 0.05) and 347 genes significantly upregulated (fold change ≥ 2, *p*-value ≤ 0.05) in lung tumor tissues compared to normal tissues. We then interrogated these data both for the enrichment of genes involved in peculiar cell functions and for the involvement of specific pathways. A GO analysis of upregulated differentially expressed genes revealed a statistically significant enrichment for genes mainly involved in the cellular process (*p*-value = 1.29 × 10^−4^), cellular component organization (*p*-value = 1.78 × 10^−4^), and developmental process (*p*-value = 0.002) (Figure 4A). The GO terms of downregulated differentially expressed genes were mainly related to the acute inflammatory response (*p*-value = 0.015) and cell adhesion (*p*-value = 0.028) (Figure 4B).

Pathways analysis individually performed on the differentially expressed genes between each normal–tumor tissue pair showed the ECM–receptor interaction pathway as the common involved pathway in all four lung tumor tissues (Table 3).

### 3.4. Analysis of Tumor Loss of Heterozygosity 

**Figure 5:**
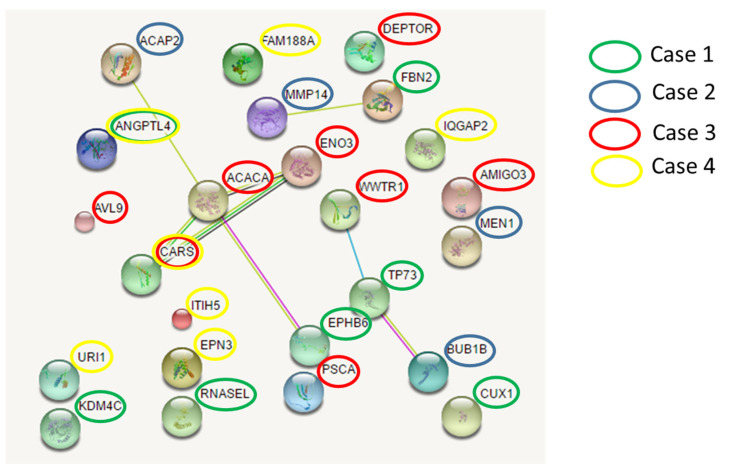
Networks among germline mutated genes. Three independent networks involving mutated genes were identified in different cases. Network nodes (colored spheres) represent proteins (empty nodes = proteins of unknown 3D structure; filled nodes = a 3D structure is known or predicted). Straight lines connecting the nodes represent protein–protein associations (blue lines = known interactions from curated databases; purple lines = known experimentally determined interactions; dark green lines = predicted interactions such as neighborhood gene; light green = predicted interactions by text mining; black lines = co-expression).

We performed the whole-exome sequencing of DNA from the four tumor tissues in order to identify potential driver genes. An analysis of the exome sequencing in DNA from blood compared to the exome sequencing in DNA from tumor tissues allowed us to identify the presence of nine variants in nine genes that were in a heterozygous state in DNA from the patient’s blood and in a homozygous state in tumor tissue (Figure 5), thus representing a possible second hit responsible for gene inactivation in this tissue.

### 3.5. Protein–Protein Interaction

To explore possible pathogenic mechanisms in lung cancer, we further investigated the existence of possible interactions among the involved cancer genes that had germline mutations. We found three networks involving mutated genes belonging to different cases (Figure 5). The main network connects the *EPHB6* gene (mutated in case 1) with the *ACACA* and *ENO3* genes (mutated in case 3), *CARS* gene (mutated in case 3 and 4), and *ACAP2* gene (mutated in case 2). Overall, each patient had deleterious germline variants in genes belonging to this network. 

**Figure 6:**
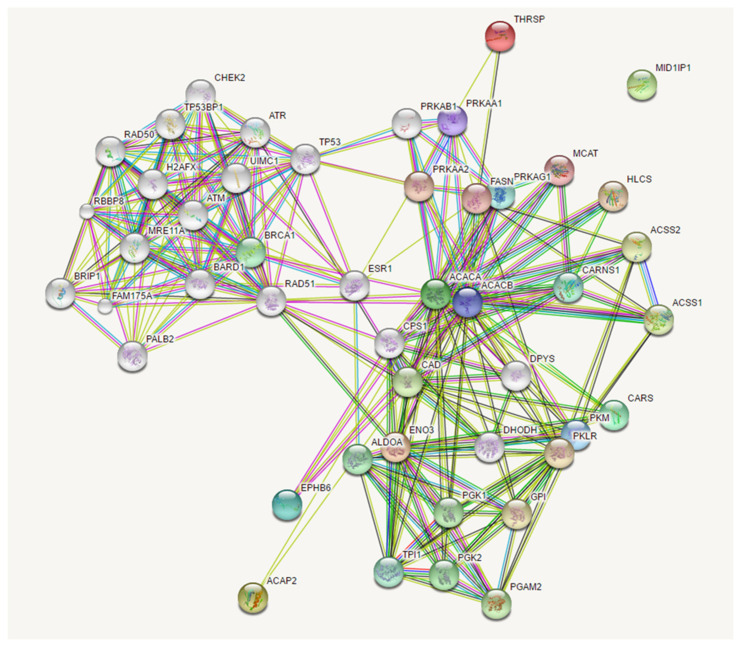
Expanded network among *EPHB6*, *ACACA*, *ENO3*, *CARS*, and *ACAP2* genes. Network nodes (colored spheres) represent proteins (empty nodes = proteins of unknown 3D structure; filled nodes = a 3D structure is known or predicted). Straight lines connecting the nodes represent protein–protein associations (light blue lines = known interactions from curated databases; purple lines = known experimentally determined interactions; dark green lines = predicted interactions such as neighborhood gene; light green = predicted interactions by text mining; black lines = co-expression; dark blue lines = gene co-occurrence; red lines = gene fusion).

Expanding the analysis of this network with a threshold of 20 interactors, we identified associations between the mutated genes in our patients with many genes involved in cancer development. In particular, the network clustered in two relevant groups, the former involving PRKA interactors, which comprised our mutated genes, and the latter involving RAD51 interactors (Figure 6).

## 4. Discussion

In this study, we used an integrative approach of next-generation sequencing technologies to dissect the genetic susceptibility to lung cancer in non-smoker patients for the identification of a genetic profile that could be predictive of the individual risk for lung cancer. The whole-exome sequencing technique, coupled with a model of cases and controls deriving from the same kindred, demonstrated that each patient has a combination of an average of 10 (range 7–14) deleterious “private” germline variants in tumor-predisposing genes. These mutations were absent in unaffected sibs. 

In addition to performing a genome-wide analysis in the search of oligogenic signatures that could differentiate affected from non-affected siblings, we also analyzed, in the same patients, the RNA-seq data in tumor specimens, comparing tumoral vs non-tumoral tissue. Using this approach, we confirmed the potential functional effect of most of the identified variants with an average of 6 (range 4–8) variants per patients. In particular, we distinguished three class of alterations: (1) variants associated with the downregulation of the gene in lung tumoral tissue compared to non-tumoral tissue, indicating the presence of a “second hit” in a putative tumor suppressor gene; (2) variants associated with upregulation in tumoral compared with non-tumoral tissue of the gene that could represent a likely oncogene; and (3) variants associated with downregulation in both tumoral and non-tumoral tissue, indicating possible mechanisms for transcript instability. 

Concerning oncogenic genes, the comparison of RNA-seq data in the tumoral vs non-tumoral tissue showed the involvement of genes belonging to molecular localization, cell movement, and cellular component assembly pathways among the upregulated genes, while, among the downregulated genes, the mainly involved pathways were cell localization, transport, negative regulation of cellular processes, and response to stress. These results seem to suggest that cancer cells miss proper intracellular molecular localization and are more resistant to stress. Comparing differentially expressed genes in matched tissue pairs, we showed the involvement of the ECM–receptor pathway in all four pairs of siblings. Similar findings have already been reported in prostate cancer [32]. Since the pathways analysis showed the ECM–receptor interaction pathway as the common involved pathway in all four lung tumor tissues, we also found that there is a relationship between this pathway and 7 of the 17 variants associated with an effect at the RNA level. The *WWTR1* in its Wwtr1/Yap Hippo pathway is known to play an essential role in the mechanosensing of alterations in cell rigidity and in the extracellular matrix (ECM) [33]. The *ANGPTL4* overexpression decreases the mRNA levels of ECM-related genes [34], the Fibrillin 2 (FBN2) is a glycoprotein of the elastin-rich ECM, being a ubiquitous glycoprotein that self-polymerize into filamentous microfibrils and is critical for ECM formation and remodeling [35]. The *MMP-14* is the driving force behind the ECM destruction during cancer invasion, and metastasis also influences both intercellular and cell–matrix communication by regulating the activity of several plasma-membrane-anchored and extracellular proteins [36]. Interestingly, *RNASEL* regulates the matrix metalloproteinases activities remodelling the ECM and plays a critical role in cell migration, invasion, tissue metastasis, and impact tumor progression [37]. The ancestor role of *TP73* was associated with the tissue organizer, but, in the developing ovary, p73 regulates a set of genes involved in ECM interactions, and other biological processes required for proper follicle development [38]. Finally, the role of IQGAP2 in receptor signaling has recently emerged, although the functions of the LGR4:IQGAP2 complex remain unidentified. However, the best known and most studied isoform of the known three, IQGAP1, promote the degradation of the ECM by matrix metalloproteinases, thereby coordinating cell invasion [39].

Network analysis concerning the identified cancer susceptibility genes showed three networks that were shared by different patients, suggesting a possible common path for private oligogenic signatures. The main network involving genes mutated in all four patients (*ACACA*, *ACAP2*, *ENO3*, *EPHB6*, and *CARS*) connected the candidate mutated genes with the group of *RAD51* (RAD51 recombinase) and *PRKA* interactors. *RAD51* encodes for a key recombinase that seems to be essential for homologous recombination and DNA repair [40]. It interacts with a large number of proteins involved in double-strand DNA breaks and in the cell cycle with important implications in tumorigenesis, such as *BRCA1* and *TP53* (as reviewed in [41]). *PRKA* encodes for the catalytic subunit of AMP-activated protein kinase (AMPK) that is a cellular energy sensor that maintains energetic homeostasis [42] and has been suggested as a novel target for anticancer therapy since its activation determines a reduction in mRNA translation and protein synthesis [43]. Figure 5 and Figure 6 show the possible network (Figure 5) and “expanded” network (Figure 6) of proposed cooperation among the main mutated genes in the occurrence of “increased susceptibility” to NSCLC. We have previously discussed the putative role of each gene in the occurrence of lung cancer.

Our study has a number of strengths and limitations. One of the main strengths is that we used an integrative next-generation sequencing approach combining the whole-exome sequencing technique (germline and tumor DNA) with transcriptome sequencing (tumor and matched normal tissue). In addition, the original selection strategy of discordant sibs has been used. Among the limitations, variants in noncoding regions, copy number variations, genome rearrangements, and common susceptibility variants have been missed due to the study design. Secondly, the number of samples analyzed is relatively small. However, the private nature of the oligogenic susceptibility reduces the potential contribution of additional discordant sib-pairs. 

In any case, the “novelty” that present results strongly suggest is the following. Opposite to what occurs in inherited multitumoral syndromes, such as Familial Adenomatous Polyposis (FAP), where germline mutations of a single gene (i.e., APC gene) determines colon cancer in 100% of affected patients, in the present model, that is likely to occur in most frequent sporadic cancers, variable oligogenic combinations of germline mutations, that change from an individual to another, all together are responsible for the occurrence of a “susceptible” or “resistant” phenotype towards a given cancer. 

In this “omics” study, in which various next-generation technologies were used, a lot of data both in quantitative and qualitative terms for each patient were produced, and this is a clear example of how their interpretation is the keystone to uncovering the oligogenic germline signature and achieving personalized precision medicine in lung cancer. Precision medicine in lung cancer is already a reality in the metastatic stage, but studies like this must be implemented to extend this approach to all lung cancer patients at different stages. Indeed, our findings showed that private oligogenic signatures could be part of an individual way to cancer. We suggest that every patient has his/her peculiar signature of germline mutations, governing personal cancer susceptibility. This signature may play a major role in the development and growth of lung cancer, namely, in non-smoker patients, and this may therefore explain the non-heritability of the condition. In fact, lung cancer in non-smokers rarely shows familial aggregation but rather seems sporadic or occurs in phratries. These two possibilities were perfectly explained by our private oligogenic model of inheritance [24]. The proposed model may have important implications in the evaluation of individual risk for lung cancer and may lead to a “germline genetic signature”, which, in the modern era of personalized medicine, could be of benefit to the early detection of cancer.

## 5. Conclusions

In conclusion, further studies are necessary in order to confirm the present findings in larger studies. In any case, our “focused analysis” in a small number of patients could contribute to a deeper insight into the complexity of the various subtypes of lung cancer and the variable interplay among gene programs involving biological processes, which could seem apparently distinct and/or distant from each other. 

## Figures and Tables

**Table 1:** Clinical characteristics and sequencing data for lung cancer cases and their healthy sibling.

	Case 1	Sib 1	Case 2	Sib 2	Case 3	Sib 3	Case 4	Sib 4
Gender	F	M	F	F	F	F	F	M
Age at diagnosis, years	52	NA	65	NA	69	NA	55	NA
Age at sampling, years	NA	44	NA	64	NA	75	NA	54
Smoker status	Never	Never	Never	Never	Never	Never	Never	Never
Histologic type	ADCA	NA	ADCA	NA	ADCA	NA	ADCA	NA
Clinical stage	I	NA	I	NA	I	NA	II	NA
Follow-up status	Alive	Alive	Alive	Alive	Alive	Alive	Alive	Alive
Age at follow-up	53	45 ^a^	66	65 ^a^	71	75	57	54
Exome sequence data, Mbp	5.63	5.48	9.06	6.72	3.11	11.8	12.8	5.3
Number of reads, Mil	38.3	37.5	49.9	36.8	17.1	65.1	69.0	28.3

NA, not applicable. ^a^ cancer-free.

**Table 2:** Sequence variants in cancer-related genes identified by whole-exome sequencing.

Gene Symbol: Mutation	Case 1	Case 2	Case 3	Case 4	Gene Name	KEGG Pathway/Function
***ACAP2*** **:c.C976T:p.R326X**	**✓**				ArfGAP with coiled-coil, ankyrin repeat, and PH domains 2	Endocytosis/Arf6 signaling events
*ACTL6A*: c.T673A:p.S225T	✓				Actin like 6A	Chromatin organization/DNA Double-Strand Break Repair
***BUB1B*** **:c.T2609C:p.V870A**	**✓**				Budding uninhibited by benzimidazoles 1 homolog beta	Cell cycle/Mitotic function (TS)
*DDX11*: c.G814A:p.V272M	✓				DEAD/H-box helicase 11	NA/(TS)
*EPB41*::c.G1264A:p.E422K	✓				Erythrocyte membrane protein band 4.1	Tight junction/Sertoli–Sertoli Cell Junction dynamics
***MEN1*** **: c.G301A:p.V101I**	**✓**				Menin 1	Transcriptional misregulation in cancer/Putative TS associated with a syndrome known as multiple endocrine neoplasia type 1
***ACACA*** **:c.C1948T:p.R650W**		**✓**			Acetyl-Coenzyme A carboxylase alpha	Fatty acid biosynthesis, pyruvate metabolism, propanoate metabolism, and insulin signaling pathway
***AMIGO3*** **: c.C669A:p.C223X**		**✓**			Adhesion molecule with Ig like domain 3	NA
*AVL9*: c.37_38del:p.R13fs		✓			AVL9 cell migration associated	NA/Late secretory pathway protein AVL9 homolog
*CTBP2*: c.C2149T:p.R717C		✓			C-terminal binding protein 2	Wnt signaling, notch signaling, and pathways in cancer
*CTSZ*:c.G358A:p.V120M		✓			Cathepsin Z	Lysosome/Apoptosis (candidate O)
***DEPTOR*** **: c.A631T:p.R211X**		**✓**			DEP domain containing MTOR-interacting protein	mTOR signaling pathway/
***ENO3*** **: c.C642G:p.Y214X**		**✓**			Enolase 3	Glycolysis, gluconeogenesis, and RNA degradation/possible TS in lung cancer (17p13.3)
*GRM1*:c.C2185A:p.P729T		✓			Glutamate receptor, metabotropic 1	Calcium signaling pathway, neuroactive ligand–receptor interaction, and gap junction
*MYO10*: c.C5690T:p.S1897F		✓			Myosin X	Fc gamma R-mediated phagocytosis/Epithelial adherens junctions, innate immune system, and RhoGDI (Putative O)
*PFKP*:c.G311A:p.R104Q		✓			Phosphofructokinase, platelet	Glycolysis, gluconeogenesis, pentose phosphate pathway, fructose and mannose metabolism, and galactose metabolism
***PSCA*** **:c.G326A:p.W109X**		**✓**			Prostate stem cell antigen	NA/Overexpressed in prostate cancer
*ROCK1*:c.C727T:p.P243S		✓			Rho-associated, coiled-coil containing protein kinase 1	Chemokine signaling pathway, vascular smooth muscle contraction, Wnt signaling pathway, TGF-beta signaling pathway, axon guidance, focal adhesion, leukocyte transendothelial migration, and regulation of actin cytoskeleton/cytoskeleton remodeling
***WWTR1*** **:c.1199_1200insTTTA:p.L400_X401delinsLX**		**✓**			WW domain containing transcription regulator 1	Hippo signaling pathway/Gene Expression (TS)
***CARS*** **:c.G775A:p.G259S**		**✓**	**✓**		Cysteinyl-tRNA synthetase	Aminoacyl-tRNA biosynthesis/Localized in an important tumor-suppressor gene region (11p15.5)
***ANGPTL4*** **:c.637delC:p.P213fs**			**✓**		Angiopoietin-Like 4	PPAR signaling pathway. Also known as peroxisome proliferator-activated receptor (PPAR). PPAR activates gene expression.
*CUX1*: c.2413dupC:p.G804fs			✓		Cut like homeobox 1	NA/FGFR1 mutant receptor activation (TS)
*EPHB6*: c.840delC:p.S280fs			✓		EPH receptor B6	Axon guidance/(TS)
***FBN2*** **:c.G3883A:p.D1295N**			**✓**		Fibrillin 2	NA/ ERK Signaling, and degradation of the extracellular matrix (TS)
*GANAB*: c.C583T:p.R195C			✓		Glucosidase II alpha subunit	N-Glycan biosynthesis/Metabolism (TS)
*KDM4C*:c.3110delG:p.S1037fs			✓		Lysine demethylase 4C	NA/Involved in signal transduction, signaling by Rho GTPases, and chromatin organization (Putative O)
***MMP14*** **:c.C609A:p.Y203X**			**✓**		Matrix metallopeptidase 14	GnRH signaling pathway/Cell adhesion_ECM remodeling
*PTPN23*:c.G4189T:p.G1397C			✓		Protein tyrosine phosphatase non-receptor type 23	Involved in the regulation of small nuclear ribonucleoprotein assembly and pre-mRNA splicing (within a putative tumor suppressor region)
***RNASEL*** **: c.G793T:p.E265X**			**✓**		Ribonuclease L	Immune system/Mutations in this gene have been associated with predisposition to prostate cancer
***TP73*** **:c.G749T:p.G250V**			**✓**		Tumor protein p73	p53 signaling pathway, neurotrophin signaling pathway/cell cycle (TS)
*ESRRA*: c.C1162T:p.L388F			✓	✓	Estrogen-related receptor alpha	NA/Nuclear receptor transcription pathway (O)
*ESRRA*: c.C1165T:p.R389C			✓	✓	Estrogen-related receptor alpha	NA/Nuclear receptor transcription pathway (O)
*ABHD5*:c.G341T:p.R114L				✓	Abhydrolase domain containing 5	Regulation of lipolysis in adipocytes/Metabolism of lipids and lipoproteins
*ACAD9*:c.G976A:p.A326T				✓	Acyl-CoA dehydrogenase family member 9	Mitochondrial biogenesis/Respiratory electron transport
*EPHA7*: c.A2009C:p.Q670P				✓	EPH receptor A7	Axon guidance
*EPN3*:c.879delA:p.L293fs				✓	Epsin 3	Endocytosis/Promoting senescence (O)
*FAM188A*:c.1107delT:p.F369fs				✓	Family with sequence similarity 188 member A	NA (Novel TS in NSCLC)
***IQGAP2*** **:c.G1135C:p.E379Q**				**✓**	IQ motif containing GTPase activating protein 2	Regulation of actin cytoskeleton
*ITIH5*: c.1063delG:p.D355fs				✓	Inter-alpha-trypsin inhibitor heavy chain family member 5	NA/RHO GTPase effectors (TS)
*PSAT1*:c.G511C:p.A171P				✓	Phosphoserine aminotransferase 1	Glycine, serine, and threonine metabolism, vitamin B6 metabolism/metabolism of amino acids and derivatives
***URI1*** **:c.G1303T:p.E435X**				**✓**	URI1, prefoldin like chaperone	NA/Scaffolding protein with roles in ubiquitination and transcription (Putative TS)

In bold: mutations associated with an effect at RNA level. NA, not applicable.

**Table 3:** Results of pathways analysis on deregulated genes in tumor tissues compared with normal tissues.

	Case 1		Case 2		Case 3		Case 4	
KEGG Pathway	*p*-Value	Count	*p*-Value	Count	*p*-Value	Count	*p*-Value	Count
ECM–receptor interaction	9.50 × 10^−8^	22	1.40 × 10^−6^	17	5.20 × 10^−5^	20	2.60 × 10^−3^	13
Focal adhesion	4.20 × 10^−3^	25	1.80 × 10^−4^	23	3.10 × 10^−2^	26		
ABC transporters	8.30 × 10^−3^	9						
Integrin signaling pathway	3.70 × 10^−2^	26						
Tight junction			2.40 × 10^−2^	13				
Cell adhesion molecules (CAMs)			4.70 × 10^−2^	12				
Calcium signaling pathway					3.90 × 10^−2^	23		

## Data Availability

The original contributions presented in the study are included in the article/Supplementary Materials, further inquiries can be directed to the corresponding author.

## Supplementary Materials

The following supporting information can be downloaded at: https://www.mdpi.com/article/10.3390/cancers16162887/s1, Supplementary Table S1: RNA-seq data summary.
