# Supplementary material for: The Personalized Inherited Signature Predisposing to Non-Small-Cell Lung Cancer in Non-Smokers
Source: Cancers (Basel). 2024 Aug 20;16(16):2887. doi: 10.3390/cancers16162887 (PMC11352340; doi:10.3390/cancers16162887)
Supplement: Supplementary file 1 [file cancers-16-02887-s001.zip › cancers-3126408-supplementary.pdf]

Supplementary Table S1. RNA-seq data summary.

**Sample**	**Tissue type**	**Yield (Mbases)**	**Total reads**	**% of ≥ Q30 Bases**
**Case 1**	Normal	7,437	73,630,364	90.08
	Tumor	7,285	72,132,496	91.41
**Case 2**	Normal	8,349	82,663,850	90.71
	Tumor	6,710	66,438,868	91.04
**Case 3**	Normal	7,040	69,701,750	91.06
	Tumor	8,137	80,567,970	91.42
**Case 4**	Normal	6,819	67,516,206	91.37
	Tumor	7,905	78,268,894	91.46
